# Importance of Attenuation Correction (AC) for Small Animal PET Imaging

**DOI:** 10.3390/diagnostics2040042

**Published:** 2012-10-09

**Authors:** Henrik H. El Ali, Rasmus Poul Bodholdt, Jesper Tranekjær Jørgensen, Rebecca Myschetzky, Andreas Kjaer

**Affiliations:** Cluster for Molecular Imaging, Faculty of Health Sciences & Department of Clinical Physiology, Nuclear Medicine & PET, Rigshospitalet, University of Copenhagen, Blegdamsvej 3, Copenhagen DK-2200, Denmark; E-Mails: rasmuspoul@sund.ku.dk (R.P.B.); jtran@sund.ku.dk (J.T.J.); rebeccam@sund.ku.dk (R.M.); akjaer@sund.ku.dk (A.K.)

**Keywords:** attenuation correction, small animal, PET imaging, MicroPET, molecular imaging

## Abstract

The purpose of this study was to investigate whether a correction for annihilation photon attenuation in small objects such as mice is necessary. The attenuation recovery for specific organs and subcutaneous tumors was investigated. A comparison between different attenuation correction methods was performed. *Methods*: Ten NMRI nude mice with subcutaneous implantation of human breast cancer cells (MCF-7) were scanned consecutively in small animal PET and CT scanners (MicroPET^TM^ Focus 120 and ImTek’s MicroCAT^TM^ II). CT-based AC, PET-based AC and uniform AC methods were compared. *Results:* The activity concentration in the same organ with and without AC revealed an overall attenuation recovery of 9–21% for MAP reconstructed images, *i.e.*, SUV without AC could underestimate the true activity at this level. For subcutaneous tumors, the attenuation was 13 ± 4% (9–17%), for kidneys 20 ± 1% (19–21%), and for bladder 18 ± 3% (15–21%). The FBP reconstructed images showed almost the same attenuation levels as the MAP reconstructed images for all organs. *Conclusions:* The annihilation photons are suffering attenuation even in small subjects. Both PET-based and CT-based are adequate as AC methods. The amplitude of the AC recovery could be overestimated using the uniform map. Therefore, application of a global attenuation factor on PET data might not be accurate for attenuation correction.

## 1. Introduction

Small animal PET imaging is frequently used in biomedical research and plays a key role in the studies of biodistribution and pharmacokinetics of new tracers. Small animal PET imaging is also a powerful tool for studying the response of new therapy methods. Small animal imaging has gained recognition as a transitional pathway to human molecular imaging [1]. Murine models in everyday practice are ideal subjects due to rapid breeding with low cost, well developed transgenic models and the ability of modelling different human diseases. The accuracy of small animal PET imaging can suffer from degradation factors that are related to the photon interactions in matter. Annihilation photon will interact with tissue and other materials as they travel through the body. Quantitative positron emission tomography (PET) requires different types of correction methods where attenuation correction (AC) is an important one [2]. The magnitude of attenuation can mathematically be expressed by the exponential equation:

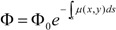
(1)
where Φ_0_ and Φ are the incident and the transmitted photon fluences (number of photon/unit area) and *ds* is a differential thickness of the tissue where the beam of photons passes through the body along the path *S*. The parameter *µ* is the linear attenuation coefficient which is the probability that the photon will undergo an interaction while passing through a unit thickness of tissue. The linear attenuation coefficient is expressed in cm^−1^. If *µ* is known, the photon attenuation in the material can be recovered. Therefore, the determination of accurate *µ* maps can determine how well the attenuation correction performs.

The magnitude of attenuation varies depending on the photon energy, the type and the size of tissue. Methods for compensation of the photon attenuation such as uniform attenuation map, CT-based attenuation and PET-based attenuation can all be used in PET imaging [3,4,5]. 

In humans, it is acknowledged that AC should always be applied in order to obtain quantitative PET data. In contrast, in small objects attenuation is assumed to be a minor problem due to small size of the animals. However, annihilation photons are subject to attenuation even in small animals such as mice. The question is whether the attenuation is an apparent problem or not and how large the attenuation recovery for the internal organs of the mouse is. The aim of our study was, to investigate whether AC is required for quantitative small animal PET in murine models. Furthermore, comparison of the quantitative accuracy of the two most commonly applied AC methods was done along with the uniform attenuation method. PET transmission method, the CT-based method and the uniform attenuation correction (*i.e*., assuming that the attenuation coefficient *µ* is constant within the animal body/water medium) are included in the comparison. 

## 2. Material and Methods

### 2.1. Animal Preparations

Ten female NMRI nude mice were obtained from Taconic Europe (Lille Skenved, Denmark) and were allowed to acclimatize for one week with *ab libitum* food and water at our animal facility. The weights of the mice ranged from ~25–30 g and the approx. dimensions of ~2 cm in the short axis and ~3 cm in the long axis. MCF-7 (human mammary cancer) cells were acquired from American Type Culture Collection (ATTC, Manassas, VA, US) and grown in DMEM standard medium supplemented with 10% fetal calf serum and 1% penicillin-streptomycin (all from Invitrogene Co., Carlsbad, CA, USA) at 37 °C in 5% CO_2_. Each mouse had approximately 10^7^ cells suspended in a mixture of 100 μL cell culture medium and 100 μL Matrigel™ (BD Biosciences, San Jose, CA, USA) injected into each flank while under anesthesia (subcutaneous injection of Hypnorm:Dormicum = 1:1 (Jansen Pharmaceutica, Beerse, Belgium and Roche, Basel, Switzerland, respectively)). The tumor sizes were ranging from 0.14 to 0.46 mL (Table 1).

**Table 1:** Mice weights and tumor sizes.

	weight [g]	tumor [mL]
mouse1	25.1	0.09
mouse2	30.2	0.14
mouse3	29.4	0.13
mouse4	30.3	0.15
mouse5	26.3	0.12
mouse6	27.1	0.12
mouse7	25.7	0.12
mouse8	25.3	0.09
mouse9	29.9	0.14
mouse10	28.2	0.13

After 3–4 weeks all animals were anesthetized by inhalation of 2.5% Sevoflurane (5% for induction) (Abbot Scandinavia AB, Solna, Sweden) mixed with 35% O_2_ in N_2_ and received approximately 10 MBq of 18F-FDG (i.v.). 

### 2.2. Imaging Experiments

All mice were scanned for 20 min, 30 min postinjection of 10 MBq FDG using a small animal PET scanner (MicroPET™ Focus 120). This was followed by a 12 min transmission scan using an external Co-57 point source attached to the system. Directly after each PET scan, a 7 min CT scan was performed intended for CT-based attenuation correction using a small animal “step and shoot” CT scanner (ImTek’s MicroCAT^TM^ II) [6,7] resulting in 468.5 million counts. The alignment of the PET and CT scan data could be accurately performed using a PET/CT interchangeable bed with three bed-fixed fiducial markers that included a CT contrast agent mixed with Ge-68 isotope.

The acquired PET data for both emission with energy window setting of 350–650 keV at 511 keV, 6 ns timing window and transmission scans with a window setting of 120–125 at 122 keV were stored in list mode. The emission list mode data post-processed to obtain 2 bytes 128 × 144 × 32 sinograms using a 3D histogramming technique [8]. The transmission sinograms were used for attenuation correction of the emission sinograms. Finally, the emission sinograms were reconstructed and resulted in 4 bytes 256 × 256 × 95 image sets with a voxel size of 0.299 × 0.299 × 0.796 mm^3^. The 2D Filtered Back Projection (2DFBP) and Maximum a Priori MAP [9,10] were used for the image reconstruction process in this work. Furthermore, the emission sinograms were corrected for dead time and decay time. Scatter correction [11,12,13,14] was not applied to the emission data to allow for a comparable ratio between the AC and the non AC images as our reconstruction software (MicroPET Manager^TM^, 2.4.1) does not allow for scatter correction of non AC data. The system was calibrated to provide an absolute activity value in a unit of Bq/ml instead of counts per voxel.

The CT data were acquired using a MicroCAT system. The acquisition time of each CT scan was approximately 7 min generating 360 projections at 360° arc. The X-ray source settings were 70 kVp, 500 μA, and 230 ms for the voltage, the current, and the exposure time, respectively. The CT projections on the 3,000 × 2,970 CCD crystal were binned by four to increase sensitivity and reduce the dataset size. The projections were reconstructed by real-time reconstruction algorithm (the Cobra toolbox) using a Shepp-Logan filter into 768 × 768 × 512 images and voxel size of 0.091× 0.091 × 0.091 mm^3^. The reconstructed CT images were uploaded using the ASIPro Toolbox (Siemens Medical Solutions, Inc., USA) for the PET/CT co-registration and the creation of the sinograms of the mass attenuation maps for CT-based attenuation correction. 

### 2.3. PET Transmission (PET-Based) AC Method

In this work a single mode acquisition was used for the PET-based AC method [15]. Following the emission scan, an external Co-57 point source attached to the MicroPET system was rotated for full coverage of the mouse for 12 min and acquiring attenuation data in list mode. The transmission list mode data were then histogrammed into sinograms. The attenuation correction sinograms (*i.e*., the mass absorption maps) were created by calculating the ratio of the blank sinograms and the transmission sinograms. The blank sinograms were obtained by a 4 h Co-57-transmission scan of an empty bed; the blank sinograms were normalized for acquisition time. The transmission sinograms were then reconstructed and compensated for scatter effect by scaling the measured linear attenuation coefficient (LAC) to the theoretical value of 511 keV. The scaled transmission images were then forward projected to create new attenuation correction sinograms. These new attenuation sinograms were then used for attenuation correction of the emission sinograms.

### 2.4. CT Transmission (CT-Based) AC Method

The CT-based AC method acquires anatomical information of the scanned subject [2,16]. The acquired data from the MicroCAT system was aligned to the acquired data from the MicroPET system using fiducial markers (Isotope Products Laboratories, Valencia, CA, USA). A manual image registration was done using the ASIPro Toolbox. The CT data were saved in MicroPET format after the co-registration with PET data involving the voxel size, the photon energy and the spatial resolution corresponding to the MicroPET scanner. The PET-matched CT images were transformed into PET attenuation maps by a forward projection. 

The CT-based AC [17] and the PET-based AC method were applied on the PET emission data. The reconstructed PET data with and without AC were then used for estimation of the attenuation in different organs (subcutaneous tumors (0.14 to 0.45 mL), kidneys and bladder). The attenuation and non-attenuation corrected images were reconstructed using MAP and FBP reconstruction techniques, respectively. 

### 2.5. Uniform Attenuation Map

The PET emission images have been used as templates to create the uniform attenuation maps (a simple global scale factor). The PET emission images were segmented to obtain the outer border of the mouse body volume. The area inside the segmented mouse volume assumed to be water and assigned an attenuation coefficient 0.096 cm^−1^ (511 keV). This attenuation map (water) was then used for attenuation correction of the PET emission data of the same mouse.

### 2.6. Statistics

For comparing means of activity concentration in a same organ using two different AC methods a paired t-test was performed. 

## 3. Results and Discussion

### 3.1. Comparison between the AC and non-AC PET Data

**Figure 1:**
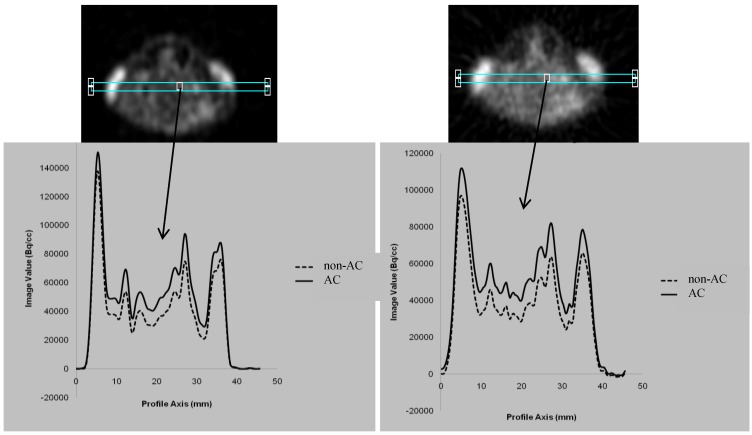
The profiles of MAP- and FBP-reconstructed images through the transaxial images are shown. The CT based attenuation correction was used. The profiles revealed that the emission images of the small animals such as mice are indeed affected by attenuation.

The acquired PET data were reconstructed with and without attenuation correction using the MAP and FBP reconstruction methods. The CT-based attenuation correction was used in this case. The comparison revealed that the estimation of the activity concentration in the small animal could be underestimated if no attenuation compensation was applied (Figure 1). The underestimation of the activity concentration was obvious in both FBP and MAP reconstructed images.

### 3.2. Attenuation Recovery versus the Choice of the Reconstruction Method

**Figure 2:**
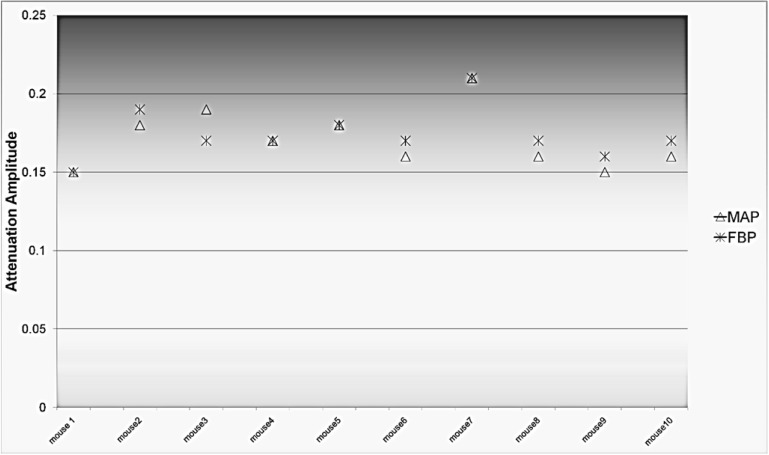
Comparison of the attenuation amplitude of the bladder for all mice using MAP and FBP reconstruction methods showed no significant variation.

To investigate if there was a significant variation in the attenuation recovery as a function of reconstruction method, 3D region of interest (3DROI:s) were drawn on both transaxial MAP reconstructed and FBP images for delineation of the mice bladders. The bladder has been chosen for this comparison to investigate how well these algorithms can deal with high activity concentration (hotspot) regions.The comparison shows that the attenuation recovery for the same organ is not dependent on the choice of the reconstruction method. The variation in the AC recovery was very small as it is shown in Figure 2.

### 3.3. Comparison between the AC methods

**Figure 3:**
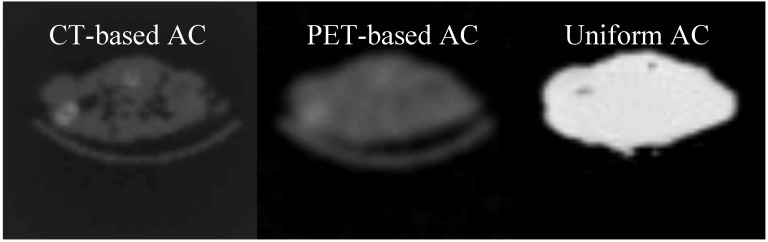
Attenuation map of one mouse using the three different AC methods: CT-based, PET-based and uniform attenuation methods.

**Figure 4:**
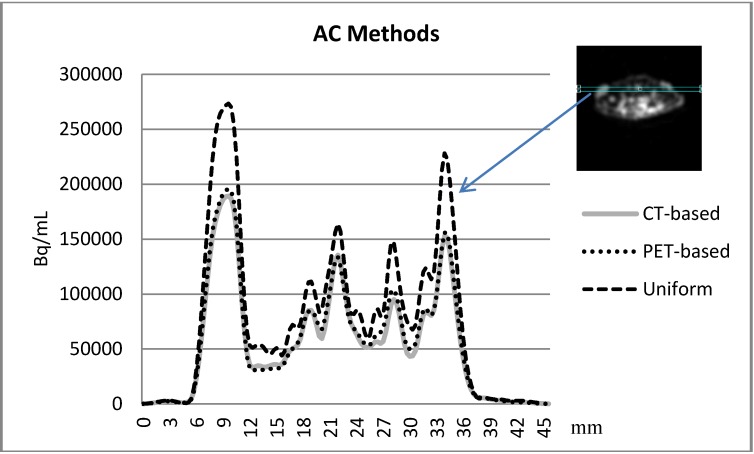
The activity profile was applied on the MAP reconstructed CT-based images PET-based and the uniform attenuation map respectively. The vertical axis of the profile is showing the activity in Bq/ml unity while the horizontal axis is the spatial distance in mm.

A visual presentation between the attenuation coefficient maps using the three different AC methods is shown in Figure 3. Figure 4 presents the comparison between the PET-based and CT-based MAP reconstructed images.

The results showed that there was no difference in the amplitude of the attenuation recovery between the PET-based and CT-based attenuation correction methods.

### 3.4. The Attenuation Recovery of the Different Organs

**Figure 5:**
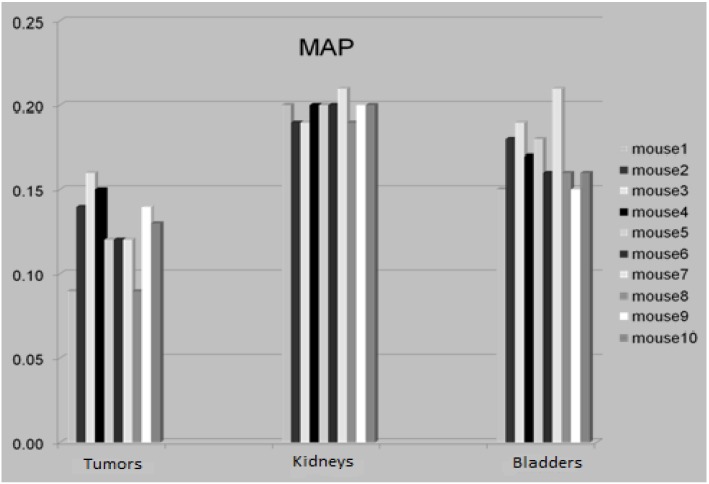
The attenuation amplitude of the tumors, kidneys and bladders respectively.

The attenuation recovery amplitudes of the tumors, kidneys and bladder of all mice were estimated using the MAP reconstruction and PET-based attenuation correction methods. This location was chosen to demonstrate a peripheral location including subcutaneous tumors plus central locations of high tracer concentration (kidneys and bladder) (Figure 5).

### 3.5. Discussion

The importance of attenuation correction (AC) for small animal PET imaging has been addressed by Chow *et al.* [2]. In that paper two phantoms (one mouse-sized and one rat-sized) and two animals (one mouse and one rat) were used for evaluating the quantitative accuracy of attenuation correction for small animal imaging. In our paper, we have included a group of mice to highlight the variation of attenuation correction recovery of different locations in the body. The bladder, kidneys and subcutaneous tumors were chosen to present different locations. 

The activity estimation could be underestimated if no attenuation compensation is applied to the emission data, albeit the subject to be scanned is very small (Figure 1).

We have used analytical (FBP) and iterative (MAP) reconstruction methods to investigate if there was a difference on the recovery of the attenuation in organs with very high activity concentration such as the bladder. Both methods showed the same amplitude of photon attenuation (Figure 2). As we expected, no difference was reported between the two reconstruction methods since neither of these methods include any scatter or attenuation compensations. 

For compensation of the photon attenuation, we compared three attenuation correction methods: PET-based AC, CT-based AC and uniform attenuation map based AC. Figure 3 shows the attenuation map obtained by using the three different AC methods. A visual inspection of the attenuation maps shows that the uniform attenuation map could overestimate the attenuation recovery due to a constant recovery factor throughout the whole body. In the uniform map, an extension of the border is leading to overestimation of the attenuation recovery near the body edges. The substantial overestimation near the edges of the tumors is probably caused due to inaccurate delineation of the border and inclusion of regions such as skin, fur or regions that would be otherwise air (~0) and are now assigning a value of 0.095 in the attenuation map. The small animal PET scanner (MicroPET), with a bore size of 12 cm and an energy window setting of 350–650 at 511 keV; 6 ns, makes the scatter fraction so low that the effect on the true events is negligible [18].

Finally, as the approximate short axis dimensions of these mice are 2 cm in diameter, it is possible to exclude everything outside that boundary when creating the borders for the uniform map (either in the sinogram or image space).

The AC overestimation at the borders using the uniform map could be reduced if accurate segmentation that delineates the soft tissue (including skin) and excludes everything (including fur) outside that boundary when creating the borders for the uniform map.

We expected that there could be some disparity in AC recovery using the PET-based and the CT-based AC methods since both methods have advantages and disadvantages.

The main advantages of the PET-based method are that the emission and transmission images are fully aligned and there is a minimum risk for subject motion and no need for the use of additional scanner.The disadvantages however could be noisy transmission images (irrespective of the length of acquisition) and inaccuracy in the scaling of the linear attenuation coefficient from that of Co-57 of 122.1 keV into 511 keV.

In comparison, the advantages of the CT-based method are the low-noise, high resolution of anatomical maps and shorter acquisition time (7 min) compared to the PET transmission (12 min) acquisition. The disadvantages of the CT-based method could however include: Inaccuracy in PET/CT co-registration due to misalignment, inaccuracy in converting tissue attenuation coefficient from an average CT energy (40–70 keV) to 511 keV, beam hardening artifacts and high radiation dose for the animals.

In the comparison between the AC methods, we attempted to minimize some of these disadvantages by using a relative strong Co-57 point source (185 MBq) and long PET transmission acquisition time for PET-based method. For the CT-based method, we insured that there were no artifacts in the CT images and insured an accurate PET/CT co-registration.

With this optimal execution of both methods, no significant difference in the attenuation amplitude using the two different AC methods was found (Figure 4).

The activity concentration in the same organ with and without AC revealed overall attenuation amplitudeof 9–21% for MAP reconstructed images, *i.e.*, SUV without AC would underestimate the true activity by this level. For subcutaneous tumors, the attenuation recovery was 13 ± 4% (9–17%), for kidneys 20 ± 1% (19–21%), and for bladder 18 ± 3 % (15–21%) (Figure 5).The variation in attenuation recovery for the different organs indicates that attenuation correction could be inaccurate if a simple global attenuation factor is applied on PET data. There was no significant difference between the PET and CT-based AC values (P < 0.01).Both methods can be considered adequate as attenuation correction methods for quantitative small animal PET imaging.

## 4. Conclusions

Even in small animals like mice, attenuation of annihilation photons has a significant impact on the underestimation of true activity concentrations of approximately 10–20%. Accordingly, it seems necessary to perform AC if quantitative PET data are needed. Both PET-based and CT-based AC methods are comparable for the attenuation compensation in PET images. The use of a simple global attenuation factor (uniform attenuation method) could lead to recovery overestimation of attenuation. We suggest that CT-based or PET-based AC should be applied for accurate quantitative small animal PET imaging; however if less accuracy is acceptable, a uniform map attenuation correction may be applied.
